# Pelvic Actinomycosis

**DOI:** 10.1155/2017/9428650

**Published:** 2017-06-08

**Authors:** Alejandra García-García, Ninfa Ramírez-Durán, Horacio Sandoval-Trujillo, María del Socorro Romero-Figueroa

**Affiliations:** ^1^Laboratory of Medical and Environmental Microbiology, Department of Medicine, Autonomous University of the State of Mexico, 50180 Toluca, MEX, Mexico; ^2^Department of Biological Systems, Autonomous Metropolitan University, Xochimilco, 04960 Mexico City, Mexico; ^3^Coordinación Delegacional de Investigación en Salud, México Poniente, Instituto Mexicano del Seguro Social, Toluca, MEX, Mexico

## Abstract

**Introduction:**

Actinomycosis is a chronic bacterial infection caused by* Actinomyces*, Gram-positive anaerobic bacteria. Its symptomatology imitates some malignant pelvic tumours, tuberculosis, or nocardiosis, causing abscesses and fistulas. Actinomycoses are opportunistic infections and require normal mucous barriers to be altered. No epidemiological studies have been conducted to determine prevalence or incidence of such infections.

**Objective:**

To analyse the clinical cases of pelvic actinomycosis reported worldwide, to update the information about the disease.

**Methods:**

A systematic review of worldwide pelvic actinomycosis cases between 1980 and 2014 was performed, utilising the PubMed, Scopus, and Google Scholar databases. The following information was analysed: year, country, type of study, number of cases, use of intrauterine device (IUD), final and initial diagnosis, and method of diagnosis.

**Results:**

63 articles met the search criteria, of which 55 reported clinical cases and 8 reported cross-sectional studies.

**Conclusions:**

Pelvic actinomycosis is confusing to diagnose and should be considered in the differential diagnosis of pelvic chronic inflammatory lesions. It is commonly diagnosed through a histological report, obtained after a surgery subsequent to an erroneous initial diagnosis. A bacterial culture in anaerobic medium could be useful for the diagnosis but requires a controlled technique and should be performed using specialised equipment.

## 1. Introduction

Actinomycosis is a chronic bacterial infection, suppurative and granulomatous in nature, caused by bacteria of the genus* Actinomyces* [1], a group of Gram-positive anaerobic bacteria that form filamentous microcolonies [2], do not form spores, measure up to 1 *μ*m diameter, and are slow-growing [1]. Actinomycosis is an uncommon condition whose symptomatology imitates some malignant pelvic tumours, tuberculosis, or nocardiosis because it spreads progressively and continuously [3]. This pathology invades tissue layers, causing the formation of abscesses and fistulae. Its diagnosis is difficult, and it results in increased morbimortality.* Actinomyces* belong to the phylum* Actinobacteria *and to the order* Actinomycetales*. Hundreds of* Actinomyces* species exist, most of which inhabit the soil. Others are associated with plants, which participate in nitrogen fixation, and a few species live in human beings as saprophytic bacteria [2]. It should be highlighted that most* Actinomyces* spp. are present in microbiota, chiefly inhabiting the oropharynx, gastrointestinal tract, and urogenital tract [3].

Actinomycoses are opportunistic chronic infections [4], as* Actinomyces* have a low potential for virulence in connection with fimbriae. Therefore, they require normal mucosal barriers to be altered through trauma, surgery, or an infection. In this way, they cross the mucosal membrane or epithelial surface [4–6]. For example, a pulmonary infection can be caused by bronchoaspiration [5, 7], or a pelvic infection can originate from the use of an intrauterine device (IUD), which can injure or perforate the mucosal membrane of the uterus and facilitate infection [3].

Currently, various clinical characteristics of actinomycosis have been described, and the bacterium has been observed in various anatomical sites (e.g., face, bones and articulations, respiratory tract, urogenital tract, digestive tract, central nervous system, skin, and soft tissue structures). The most frequent clinical form of the disease is cervicofacial actinomycosis, representing approximately 60% of all reported cases, and is associated with odontogenic infection. Other clinical types include thoracic actinomycosis, the third most common type of actinomycosis, which includes pulmonary, bronchial, and laryngeal actinomycosis [3], and abdominal actinomycosis, where the appendix, caecum, and colon are the most common sites of infection. Actinomycosis of the central nervous system is located chiefly in the cerebral abscess. Actinomycosis of the urogenital tract is the second most common clinical form of actinomycosis, and the principal clinical presentation is pelvic actinomycosis [3, 5, 8].

Pelvic actinomycosis can affect any age group, with no preference for occupation or season and is secondary to perforation or fistulation [4]. Other possible causes include bacterial vaginosis, which fosters an anaerobic environment and is associated with other microorganisms [51]; the presence of tumours [66]; and the use of IUDs [3–5]. The possibility of a contagion through oral sex has been considered because these bacteria are part of the oral cavity microbiota [72]. One possible route of dissemination is through IUDs, which fosters the growth of microorganisms through wires that are left in the exocervix. In addition, the IUD changes the carbohydrate metabolism in endometrial cells, fostering still more inflammation. Another probable route is the perineum, where the microorganisms could extend from the anus up through the cervicovaginal zone [4].

The most common aetiological agent is* Actinomyces israelii* [5, 73]. Other reported species include* A. naeslundii, A. viscosus, A. odontolyticus, A. pyogenes, A. urogenitalis, *and* A. turicensis* [72, 74, 75].

The symptoms of pelvic actinomycosis associated with the use of an IUD can imitate symptoms of gynaecological malignant tumours, uterine myoma, or adenomyosis when presenting as a genital mass without fever [3]. The infection can disseminate to the uterine tubes and can cause salpingitis and the subsequent destruction of the ovarian parenchyma [4]. Organs such as the bladder, ileocaecal (iliac fossa) and rectosigmoid region, colon, urethra, and extension to the skin have been reportedly affected in various published cases.

The diagnosis of pelvic actinomycosis is obtained using various techniques because culturing* Actinomyces *spp. presents difficulties and also depends on the skill and access to equipment necessary to perform it.

- First, the signs and symptoms of the patients are considered and can point to a possible abdominal infection, vaginitis, abscess, or possible tumour-forming process. The most common symptoms are weight loss, nonspecific abdominal or pelvic pain, breakthrough bleeding or abundant vaginal flow, and, on rare occasions, fever [3, 4, 51].
- Upon medical exploration, the affected zone is palpated to detect hard masses, and a gynaecological exam is performed to check for inflammation of the vaginal mucous membrane, yellowish secretion with a bad smell, or some visible damage to the mucous membrane [4, 51].
- In laboratory studies, it is possible to identify leucocytosis, erythropaenia, and high sedimentation rate; high values of C-reactive protein; and tumour marker values within the reference ranges or slightly elevated like Ca 125 (Alpha-fetoprotein), and cancer antigen 15–3 [3, 4, 51].
- Diagnostic images, such as computed tomography, magnetic resonance, ultrasound, X-rays, and laparoscopy are helpful, as they can be used to observe the affected zone, such as a tumour-forming mass that can induce either actinomycosis or a carcinogenic process [4, 51, 73].
- In most cases, histological visualisation of biopsy or aspirated samples is employed, where bacilli in the tissue with their typical ramifications, such as in interconnected breasts, are observed. Cervicovaginal cells are collected for Papanicolaou (Pap) staining. In both cases, they are reported as Microorganisms Similar to* Actinomyces* (MSA) [4, 51]. In many cases, the diagnosis is made* a posteriori* through a histological examination of samples obtained surgically during laparotomy or laparoscopy, but rarely in a preoperative manner. Histological studies of tissues show inflammatory changes of suppurative and granulomatous nature, connective proliferation, and sulphur granules, which have also been identified in infections caused by* Nocardia brasiliensis, Actinomadura madurae,* and* Staphylococcus aureus*. These granules are particles of yellowish colour, which, when viewed by the naked eye, are formed by groups of filamentous* Actinomyces* surrounded by neutrophils [73].
- Two methods exist for completely identifying the causal agent: culture and identification through biochemical tests and identification through sequencing of the 16S rRNA segment, which offers greater precision. Although these methods are very efficient, they are not well reported in the literature due to the conditions under which they must be performed, requiring an anaerobic culture environment and the necessary equipment, which is costly.

The usual treatment for actinomycosis consists of high and prolonged doses of penicillin G (20 million units per day) or amoxicillin for 4 to 6 weeks, followed by penicillin V (4 g per day) orally for 6 to 12 months. Clindamycin, tetracycline, and erythromycin are an alternative in cases of allergy to penicillin [4, 5]. In addition to these medicines, it has been observed that* Actinomyces* is also sensitive to third-generation cephalosporins, ciprofloxacin, trimethoprim-sulfamethoxazole, and rifampicin [4]. However, the elimination of the injured tissue and surgical drainage are necessary measures in some cases [5], and, in these patients, the duration of antimicrobial therapy could be reduced (3 months) [3].

In the review performed by Martínez et al. [74], it is mentioned that there are reports of the presence of* Actinomyces* in secretions starting from 1877, recorded by Harz. Beedham et al. found that the first reports of intrauterine actinomycosis related to IUDs appeared in the 1920s [76]. Clinical cases of pelvic actinomycosis have been reported in Africa, Oceania, Asia, Europe, and America. However, as pelvic actinomycosis is an uncommon infection, no epidemiological studies have been conducted to determine its prevalence or incidence.

## 2. Materials and Methods

We performed this analysis according to the guidelines of the Meta-Analysis of Observational Studies in Epidemiology Group (MOOSE). A systematic review of worldwide cases of pelvic actinomycosis between the years 1980 and 2014 was performed. Studies that described clinical cases of pelvic actinomycosis with a detailed diagnostic method and cross-sectional studies of cases of actinomycosis available in the PubMed, Scopus, and Google Scholar databases were included using the following keywords: Pelvic actinomycosis, Actinomycosis pélvica, “Actinomycosis prevalence”, “Prevalencia de actinomycosis”, “Actinomyces” AND “female genital tract” and combinations of these terms. The use of quotes was avoided when searching for the terms Pelvic actinomycosis and Actinomycosis pélvica in order to increase the search results.

**Figure 1:**
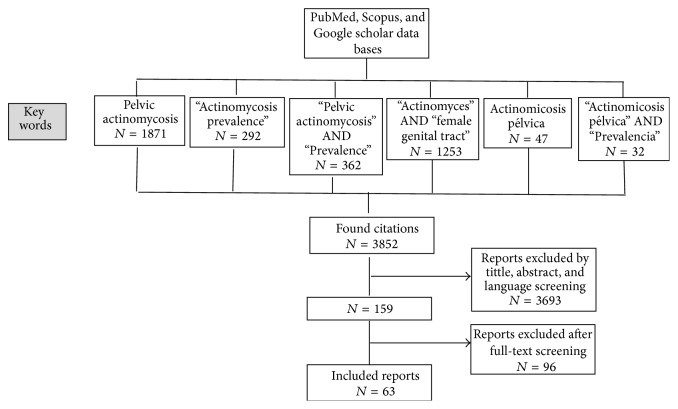
Search strategy: flow chart of literature research.

Abstracts of articles identified to be relevant for the objective of this paper were read; studies whose abstract or full text was unavailable were automatically excluded. When an abstract complied with inclusion criteria, the full text was analysed. Case reports that lacked a diagnostic method and a final diagnosis of pelvic actinomycosis were excluded. Studies published in a language that was not English, Spanish, French, or Portuguese were not included. The following information was extracted and analysed from the compiled studies: year, country, type of study, number of cases, prior use of IUD and duration, initial diagnosis, treatment, definitive diagnosis, and method of definitive diagnosis (Figure 1).

## 3. Results

The search yielded a total of 3852 studies; 3693 were excluded from the title, abstract, and language screening; 96 more were excluded for not being available in full text format and for not meeting the selection criteria when reading the full article.

A total of 63 studies including 86 case reports of pelvic actinomycosis, along with 8 cross-sectional studies of reports examining populations for cases of Microorganisms Similar to Actinomyces (MSA), were included for this review (Figure 1).

### 3.1. Cases of Pelvic Actinomycosis Reported in Africa

From the African continent, 3 articles of clinical cases were found, totalling 8 clinical cases. The majority of patients were IUD users; however in most cases the type of IUD used was not disclosed. The pathology that was first diagnosed in these cases was an ovarian tumour. The method of diagnosis that was utilised to definitively diagnose patients with actinomycosis was histopathological reporting (Table 1). The most common treatments were hysterectomy, laparotomy, and antibiotic therapy. No follow-up data was presented.

### 3.2. Cases of Pelvic Actinomycosis Reported in Oceania

From Oceania, 1 article was published that included 3 clinical cases with the following ages: 56, 70, and 37 years; two of them were copper IUD users. In the three cases, malignant lesions were initially diagnosed; the final diagnosis was performed postoperatively. Salpingo-oophorectomy along with antibiotic therapy was used in all the cases; patients fully recovered after treatment. (Table 2).

### 3.3. Cases of Pelvic Actinomycosis Reported in Asia 

Fourteen articles of 16 clinical cases came from Asia, the age of the patients ranged between 25 and 86 years, and the average age was 45.6 years (SD 15.5). The majority of patients were IUD users, with a usage time of 1 year to more than 20 years; most of the studies did not specify the type of IUD used. However, cases in nonusers were also reported, despite the well-known relationship between IUD use and pelvic actinomycosis. The most common presumptive diagnostic was malignant lesions, while, in other cases, Crohn's disease and acute peritonitis were also suspected. The most utilised diagnostic method was histological reporting after surgical interventions, which were invasive in most cases, such as hysterectomy and salpingo-oophorectomy along with antibiotic therapy. Most of the patients had a full recovery or at least a significant improvement after follow-up; only a case of renal sequelae was reported. (Table 3).

### 3.4. Cases of Pelvic Actinomycosis Reported in Europe

Twenty clinical case report articles including 39 cases of pelvic actinomycosis originated from Europe, in which ages ranged from 18 to 65 years; average age was 40 years (SD 10.4). The cases principally included female IUD users, with a usage time ranging from 1.5 to 20 years; copper and multiload IUD were the most reported; however in most studies the type of IUD is not specified. The predominant presumptive diagnosis was malignant lesion; other suspected diagnoses included Crohn's disease, acute appendicitis, endometrial infection, pelvic inflammatory disease, and abscesses. Postoperative histopathological reports were the most common definitive diagnostic methods. Other methods of final diagnosis have also been reported, such as the Pap smear, culture, API 20A biochemical assays, and 16S rRNA sequencing techniques. The most common treatments used were damaged tissue excision, laparotomy, and salpingo-oophorectomy together with antibiotic therapy. The majority of the articles do not have follow-up information, nonetheless studies reporting patient follow-up stated that they fully recovered after treatment, and there is one report of death (Table 4).

### 3.5. Cases of Pelvic Actinomycosis Reported in America

With regard to America, 16 articles with reports of 20 clinical cases exist. The ages of patients ranged between 18 and 58 years with an average age of 39.6 years (SD 9.9). All patients were IUD users except one case, and the time of device use ranged from 22 months to 33 years. Reported types of IUD include Dalkon Shield and Lippes loop. Tubo-ovarian and pelvic abscesses along with malignant lesions were the conditions with the greatest diagnostic confusion. Similar to the other summaries, the postsurgical histological reports were the most reported definitive diagnostic methods. Other methods were also utilised, such as culture, the 16S rRNA sequencing technique, haematoxylin-eosin staining microscopy (HE), and the IUD smear. Salpingo-oophorectomy and laparotomy along with prolonged antibiotic therapy were the most used therapeutic measures. After treatment most of the patients had a full or significant recovery. (Table 5).

### 3.6. Cross-Sectional Studies

Eight cross-sectional studies of reports worldwide that examine populations for cases of actinomycosis or MSA were analysed. The prevalence of pelvic actinomycosis was low. Likewise, there is a strong relationship between the use of an IUD and the presence of MSA. In this type of report, the diagnosis methods reviewed were the Pap reports. However, it is important to emphasise that what is reported in these analyses are MSA. Only 3 articles reported actinomycosis as such, and only one report completely identified the causal agent through culture and biochemical assays (Table 6).

## 4. Discussion

According to the analysis of the articles presented, Europe was the continent on which the greatest number of cases of pelvic actinomycosis was reported, followed by Asia and America. However, it is important to emphasise that this summary of information only gives us an approximation of the real epidemiology of this disease, as the cases presented in this article are only those reported. The youngest cases (18 years) are found in the European and American continents, and the oldest case (86 years) is found in the Asian continent.

Actinomycosis is an invasive infection that frequently imitates malignant processes in various anatomical zones. Pelvic actinomycosis involves one of the regions that is most often a source of diagnostic confusion. In this review, it is evident that, in many of the cases presented, an erroneous clinical diagnosis was made, confusing pelvic actinomycosis mainly with malignant lesions. Other common suspects were tubo-ovarian and pelvic abscesses and Crohn's disease. As such, as described by Kayikcioglu et al. [35] and Moniruddin et al. [77], pelvic actinomycosis should be considered in the differential diagnosis in any chronic inflammatory lesion of the viscera located in the pelvic zone to prevent a diagnostic error that could lead to unnecessary invasive treatment.

The diagnosis of pelvic actinomycosis is difficult because it does not produce characteristic disease signs or symptoms. According to what was observed in this analysis, the most utilised diagnostic method in all continents was the histopathological report, which is commonly obtained after a surgical intervention due to an initial diagnostic error. This observation was also made by Purola and Paavonen [78] and more recently by Pérez-López et al. [73]. Other highly reported methods are the Pap test, which is generally reliable, but not unequivocal, as* Actinomyces* could be confused with similar organisms. Cases in which the causal microorganism is completely identified are few, as are cases identified by culture and biochemical assays. Valour et al. [3] mentioned that culturing bacteria in anaerobic medium is the cornerstone for diagnosing actinomycosis. However, this method requires a very controlled and precise technique and specific equipment. Identification by sequencing of the 16S rRNA segment is another technique that offers greater precision. Currently other authors such as Demirezen et al. [79] report the effectiveness of using specific primers to identify the most common* Actinomyces* species from patients' swabs samples; this technique is more accurate and faster than all the previous ones; the disadvantage is the high cost of reagents and the use of special equipment. However, because of the nature of the pathology, there is no early diagnosis, because, as has already been mentioned, the presence of symptoms occurs in advanced stages of the disease.

According to the observed reports, we conclude that the presentation of symptoms in pelvic actinomycosis is observed in an advanced period of the pathology, which does not include attack to the general state of health or fever, which is oriented towards an infectious pathology. The manifestations found are occupational masses in the pelvic-abdominal cavity that force the surgical procedures to be performed, and the diagnosis is made up to the time of the histopathological study.

Pelvic actinomycosis is considered to be a rare and unusual disease, although the use of IUDs can promote its appearance. In the articles analysed, the greatest number of patients diagnosed with pelvic actinomycosis on all continents was IUD users, and the periods of use varied widely, from one year to long periods, such as 33 years. However, cases occurred mainly in users that wore IUDs for prolonged periods. Thus, based on experience and observation, it is recommended that IUDs be changed periodically to limit the occurrence of this condition. Some authors, such as Valour et al. [3], recommend changing the IUD every 5 years at a minimum, and others, such as Hernández et al. [4], recommend changes every 3 years. It should be emphasised that those cases in which patients were not IUD users were identified more recently. This observation could suggest that, despite the information that is available regarding the relationship between this condition and IUD use, the aetiology of pelvic actinomycosis could be due to other factors.

Like all review studies, the main limitation of the study was the lack of data reported, another limitation was that not all articles were open access, and there were not enough subscriptions to the respective journals to access them.

During the first reports of this disease, greater numbers of cases were observed in developed countries, but, presently, reports of cases in developing countries and regions such as the Middle East, Southwest Asia, or Latin America are more common. This change could be because, in the first decades of its observation, this condition and its aetiology were unknown and prevention was difficult. However, with the advance of technology, preventative measures directed at high-risk populations in developed countries began to be applied, as opposed to developing countries, where no such actions were taken.

## Figures and Tables

**Table 1:** Publications of cases of pelvic actinomycosis in Africa.

Definitive diagnosis	Presumptive diagnosis	Definitive method of diagnosis	Previous use of IUD	Treatment and resolution	Number of cases Age (years)	Reference
Tumour mass formation caused by MSA^*∗*^	Ovarian tumour	Histopathological report	Yes (15 years) Type: ND^*∗∗*^	Laparotomy and hysterectomy, ampicillin No date of resolution	1 56	1989, Ben Nasr et al. Tunisia [9]

Pelvic actinomycosis, MSA		Histopathological report	No = 1 Yes = 4 Type: ND	Total hysterectomy and bilateral oophorectomy Penicillin Resolution different according to patient	5 39.2 (average age)	2008, Chelli et al. Tunisia [10]

MSA	Ovarian tumour	Histopathological report; direct study of right extracted ovary	Yes Type: ND	Oophorectomy, prolonged antibiotic therapy No date of resolution	2 ND	2010, Abid et al. Tunisia [11]

*Total cases*					8	

^*∗*^MSA = microorganisms similar to *Actinomyces.*

^*∗∗*^ND = not disclosed.

**Table 2:** Publications of cases of pelvic actinomycosis in Oceania.

Definitive diagnosis	Presumptive diagnosis	Definitive method of diagnosis	Previous use of IUD	Treatment and resolution	Number of cases Age (years)	Reference
Actinomycosis organisms *Actinomyces* sp.	Ovarian cancer Malignant ovarian lesion Ovarian neoplasm	Histopathological report Histopathological report, culture	Yes = 1 Type: copper IUD No = 1 Yes = 1 Type: copper IUD	Total abdominal elective hysterectomy and bilateral salpingo-oophorectomy, penicillin, and amoxicillin Complete recovery Laparotomy and bilateral salpingo-oophorectomy with left ureterolysis, ceftriaxone, and metronidazole Complete recovery Ceftriaxone and Metronidazole, subsequent laparotomy, left salpingo-oophorectomy, penicillin, and amoxicillin Complete recovery	3 56, 70, and 37	2014, Wan et al. Australia [12]

*Total cases*					3	

**Table 3:** Publications of cases of pelvic actinomycosis in Asia.

Definitive diagnosis	Presumptive diagnosis	Definitive method of diagnosis	Previous use of IUD	Treatment and resolution	Number of cases Age (years)	Reference
Actinomycotic abscesses (sulphur granules)	Sigmoid colon cancer and tumour in left ovary	Histopathological report	Yes (1 year) Type: ND^*∗∗*^	Segmented resection of the sigmoid colon, elimination of the left distal ureter, the left ovary and Fallopian tube, ampicillin, and amoxicillin Complete recovery	1 36	1995, Kim et al. South Korea [13]

MSA^*∗*^	Crohn's disease or ovarian cancer or pelvic abscess associated with the IUD	Histopathological report of the ovary	Yes (14 years) Type: ND	Laparotomy, total hysterectomy, bilateral salpingo-oophorectomy, and anterior resection No date of resolution	1 45	2009, Lim et al. Korea [14]

MSA	Tumour in the appendix	Histopathological report	ND	ND	1 50	2010, Lee et al. South Korea [15]

Pelvic actinomycosis (*A. israelii*)	ND	Sonography-guided transvaginal needle aspiration	Yes (4 years) Type: multiload copper IUD	Drainage, penicillin, and amoxicillin Complete recovery	1 38	1996, Anteby et al. Israel [16]

*Actinomyces*, pelvic actinomycosis	Peritoneal carcinomatosis	Schiff and Grocott-Gomori acid tests	Yes (10 years) Type: ND	Incomplete tumourectomy, ileal resection, partial cystectomy, colostomy and bilateral ureterocutaneostomy, and penicillin Significant improvement	1 43	1999, Maeda et al. Japan [17]

Pelvic actinomycosis	Pelvic actinomycosis	Cervical Papanicolaou	Yes (21 years) Type: ND	Ampicillin Almost complete recovery	1 51	2007, Nozawa et al. Japan [18]

Pelvic actinomycosis	Ovarian malignancy	Gomori methenamine staining histopathology	No	Hysterectomy with bilateral salpingo-oophorectomy No date of resolution	1 74	2012, Ikeda and Kato Japan [19]

Puncture pyometra caused by *Actinomyces*	Puncture pyometra	Microscopic examination, Gram staining of the uterus and intraperitoneal pus, and culture	No	Emergency abdominal hysterectomy and bilateral salpingo-oophorectomy, cefmetazole, and meropenem No date of resolution	1 86	2013, Hagiya Japan [20]

Inflammation caused by MSA	Acute peritonitis due to perforated viscera	Histopathological report of the abdominal wall	Yes (20 years) Type: ND	Laparotomy, resection, and penicillin Complete recovery	1 50	2008, Devendra and Chen Singapore [21]

MSA	Pelvic actinomycosis	Papanicolaou, cervical culture and culture of IUD (without being able to be isolated), and histopathological report	Yes (15 years) Type: ND	Laparotomy, amoxicillin, and penicillin Significant recovery	1 40	2010, Fu and Tasi Taiwan [22]

MSA, *Actinomyces* spp.	Ovarian cancer	Histopathological report and culture of purulent material	No	Laparotomy, hysterectomy, penicillin, and streptomycin Complete recovery	3 25, 31, and 35	2010, Munjal et al. India [23]

Endometrial actinomycosis	ND	Histopathological report of endometrial samples	No	Augmentin and amoxicillin No date of resolution	1 52	2012, Sharma et al. India [24]

Ovarian actinomycosis	Ovarian cancer	Histopathological report	No	Laparoscopy, hysterectomy with salpingo-oophorectomy, and penicillin Total recovery	1 39	2013, Vijaya et al. India [25]

Pelvic actinomycosis, *Actinomyces*	Ovarian cancer	Histopathological report	No	Total hysterectomy with bilateral salpingo-oophorectomy No date of resolution	1 35	2013, Chalageri et al. India [26]

*Total cases*					16	

^*∗*^MSA = microorganisms similar to *Actinomyces.*

^*∗∗*^ND = not disclosed.

**Table 4:** Publications of cases of pelvic actinomycosis in Europe.

Definitive diagnosis	Presumptive diagnosis	Definitive method of diagnosis	Previous use of IUD	Treatment and resolution	Number of cases Age (years)	Reference
*Actinomyces*	Crohn's disease	Histopathological report of purulent material	Yes (20 months) Type: ND^*∗∗*^	Laparotomy, penicillin, and fusidic acid Complete recovery	1 19	1985, Spickett and Kipping England [27]

MSA^*∗*^	Ovarian cancer with metastasis	Histopathological report	Yes (4 years) Type: ND	Total abdominal hysterectomy and bilateral salpingo-oophorectomy, and penicillin No date of resolution	1 37	1997, Kirova et al. France [28]

Actinomycosis	ND	Papanicolaou	Yes (15 years) Type: copper IUD	Amoxicillin/clavulanic acid and ofloxacin Complete recovery	1 57	2013, Rajaonarison et al. France [29]

*Actinomyces israelii*	ND	Culture of drained sample	Yes (8 years) Type: copper IUD	Drainage of abscess (colpoceliotomy), coamoxiclav Complete recovery	1 53	2013, Tholozan et al. France [30]

MSA Inflammation caused by *Actinomyces*	ND	Histopathological report	Yes (19 and 7 years) Type: ND	Laparotomy, preoperative biopsy, resection of the tumour, resection of the necrotised tissues and partial cystectomy, hysterectomy, bilateral salpingo-oophorectomy, penicillin, and amoxicillin Complete recovery	2 48 and 52	2000, Pérez García et al. Spain [31]

MSA	Malignant tumour formation	Histopathological report	ND	Laparotomy, resection of the central part of the epiploon and tumour formation, penicillin, and amoxicillin Significant improvement	1 30	2009, García Martínez et al. Spain [32]

MSA	ND	Histological cervicovaginal observation and histopathological report	Yes Type: ND	Laparotomy, penicillin, and amoxicillin Complete recovery	2 33 and 35	2003, Bergenhenegouwen et al. Holland [33]

*Actinomyces*	ND	Aspirate study	Yes Type: ND	ND	1 45	2005, Lely and Van Es Holland [34]

MSA	ND	Histopathological report	Yes (4–9 years) Type: copper IUD	Penicillin, bacampicillin Complete recovery	5 32, 35, 44, 44, and 52	2005, Kayikcioglu et al. Turkey [35]

Actinomycosis, MSA	Tumour formation or abscess in ovary	Histopathological report of the ovary	Yes = 2 (15 and 6 years) No = 1 Type: ND	Sulbactam-ampicillin, penicillin and ceftriaxone, laparotomy, drainage of abscesses salpingo-oophorectomy, and hysterectomy Complete recovery	3 32, 45, and 55	2009, Onal et al. Turkey [36]

MSA	ND	Histopathological report	Yes (8 years) Type: multiload copper IUD	Extraction of a mass in the internal walls of the abdomen, penicillin Complete recovery	1 48	2010, Carkman et al. Turkey [37]

Damage in the organs adjacent to the irregular mass, MSA	ND	Histopathological report	Yes (16 years) Type: ND	Laparotomy, total abdominal hysterectomy, bilateral salpingo-oophorectomy, and penicillin Complete recovery	1 48	2000, Yegüez et al. Turkey [38]

Actinomycosis	Pelvic inflammatory disease, rectal tumour	Histopathological report	Yes (12 years) Type: copper IUD	Laparotomy, hysterectomy, bilateral salpingo-oophorectomy, appendectomy, lower anterior resection, Hartmann colostomy, penicillin, and amoxicillin Complete recovery	1 44	2012, Yilmaz et al. Turkey [39]

Actinomycosis		Necropsy	Yes (20 years) Type: ND	Death due to sepsis	1 49	2007, Grabiec et al. Poland [40]

MSA	Acute appendicitis, fistulisation in abdominal wall	Histopathological report	No	Laparotomy, right ileocolic resection with anastomosis of the ileotransverse colon, and amoxicillin No date of resolution	1 46	2008, Pitot et al. Belgium [41]

*Actinomyces *spp.	Carcinoma	Purulent material culture, histopathological report	Yes (3 years) Type: ND	Right hemicolectomy, antibiotic therapy Complete recovery	1 35	2009, Čolović et al. Serbia [42]

MSA, pseudoactinomycotic radiate granules	Endometrial infection, ovarian abscess, and both	Histopathological report	Yes Type: ND	5 endometrial biopsies and 1 piece of hysterectomy No date of resolution	6 ND	2009, Boyle and McCluggage North Ireland [43]

*Actinomyces*	Muscular neoplasia	Postsurgical histopathological report of samples from the abdominal wall abscess	Yes Type: ND	Laparotomy, adhesiolysis, complete excision of the mass with extensive damage to the anterior abdominal wall, and antibiotic therapy No date of resolution	1 47	2010, Acquaro et al. Italy [44]

Anogenital actinomycosis, *Actinomyces turicensis*	Perianal abscesses, pilonidal cyst, and gas gangrene	API 20A biochemical assays and 16S rRNA sequencing technique	ND	ND	7 18, 18, 28, 23, 28, 33, and 65	2010, Chudáčková et al. Czech Republic [45]

Actinomycosis	Tumoural process in pelvis	Histopathological report	Yes Type: multiload copper IUD	Cystoscopy, penicillin, and Duomox No date of resolution	1 42	2012, Maxová et al. Czech Republic [46]

*Total cases*					39	

^*∗*^MSA = microorganisms similar to *Actinomyces.*

^*∗∗*^ND = not disclosed.

**Table 5:** Publications of cases of pelvic actinomycosis in America.

Definitive diagnosis	Presumptive diagnosis	Definitive method of diagnosis	Previous use of IUD	Treatment and resolution	Number of cases Age (years)	Reference
*Actinomyces israelii*	Tubo-ovarian abscess	Histopathological report and culture	Yes (4 years) Type: Dalkon Shield IUD	Laparotomy, hysterectomy, bilateral salpingo-oophorectomy, and penicillin Complete resolution	1 29	1980, McLeod et al. United States [47]

Actinomycotic tubo-ovarian abscess	Tubo-ovarian abscess or malignant tumour	Histopathological report	Yes Type: ND^*∗∗*^	Antibiotic therapy, tumourectomy, and right salpingo-oophorectomy No date of resolution	1 29	1982, Kelly and Aaron United States [48]

*A. naeslundii*	Pelvic abscess	Microscopic observation of the IUD and culture	Yes (10 years) Type: Dalkon Shield IUD	Antibiotic therapy hysterectomy, bilateral salpingo-oophorectomy Complete recovery	1 39	1985, Bonnez et al. United States [49]

Sulphur granules; *Actinomyces israelii*; actinomycotic pelvic abscess secondary to IUD involving the bladder, sigmoid colon, left ureter, liver, and superior abdominal wall	ND	Histopathological report and culture	Yes (15 years) Type: Lippes loop IUD	Percutaneous drainage and prolonged antibiotic therapy No date of resolution	1 41	1996, Hochsztein et al. United States [50]

Actinomycotic granules, tubo-ovarian abscess	Abdominal tumour secondary to colon cancer	Laparotomy	Yes Type: copper IUD	Laparotomy, en bloc resection that included compromised abdominal wall, right hemicolectomy, hysterectomy, bilateral salpingo-oophorectomy, partial sigmoidectomy, and penicillin Complete recovery	1 47	1999, Mesa-Castillo et al. Colombia [51]

MSA^*∗*^	ND	Histopathological report	Yes (9 and 3 years) Type: ND	Laparotomy, hysterectomy, oophorectomy, penicillin, and amoxicillin Significant improvement	2 38 and 23	2006, Urbina et al. Colombia [52]

*Actinomyces *sp.	Bilateral cystic teratoma	Histopathological report	No	Laparotomy, bilateral salphingo-oophorectomy, and penicillin Significant improvement	1 47	2001, Burlando et al. Argentina [53]

Tubo-ovarian actinomycosis, MSA	Tumour formation	Histopathological report of the ovary	Yes (8 years) Type: ND	Oophorectomy, right salpingectomy, and amoxicillin No date of resolution	1 41	2005, Vispo et al. Argentina [54]

Actinomycosis, MSA	Vesical tumour	Histopathological report	Yes (33 years) Type: ND	Penicillin Progressive improvement	1 58	2003, Alegría et al. Chile [55]

Pelvic actinomycosis, sulphur granules	Pelvic or neoplastic actinomycosis of the colon or ovary	IUD swab	Yes (27 years) Type: Lippes loop IUD	Penicillin and amoxicillin Significant improvement	1 54	2013, Daniels et al. Chile [56]

MSA	ND	Histopathological report of the right ovary	Yes (22 years) Type: ND	Laparotomy to drain purulent material, hysterectomy with bilateral salpingo-oophorectomy, and penicillin Significant improvement	1 48	2004, López-Cervantes et al. México [57]

MSA Actinomycotic granuloma	Uterine myomatosis	Histopathological report	Yes (10, 10, and 4 years) Type: ND	Laparotomy, hysterectomy, salpingo-oophorectomy, and penicillin Significant improvement	3 36, 37, and 39	2005, Olivera-Reynada et al. Mexico [58]

Coinfection by *Neisseria gonorrhoeae *and *Actinomyces naeslundii*	ND	Culture of UID and vaginal exudate	Yes (22 months) Type: copper IUD	Surgical excision of the appendix, bilateral salpingectomy No date of resolution	1 36	2013, Eiros-Bouza et al. Mexico [59]

Urachal actinomycosis, “sulphur granules”	Carcinoma	Histopathological report	ND	Partial cystectomy Complete recovery	1 46	2013, Alfonso et al. Mexico [60]

*Actinomyces *spp.	Cyst in the left ovary and abscess in the iliac fossa	Purulent material, histopathological report	Yes (2 years) Type: ND	Laparotomy, cefotaxime, metronidazole, and penicillin Gradual recovery	1 18	2004, Mejia et al. Mexico [61]

*Actinomyces urogenitalis*	ND	Microscopic observation of the IUD, sequencing of the 16S rRNA gene	Yes Type: ND	Oral amoxicillin Complete recovery	1 38	2006, Elsayed et al. Canada [62]

Ovarian actinomycosis	Left tubo-ovarian abscess	Haematoxylin-eosin (HE) staining microscopy	No	Exploratory laparotomy, unilateral oophorectomy, and penicillin Complete recovery	1 49	2013, Bes et al. Brazil [63]

*Total cases*					20	

^*∗*^MSA = microorganisms similar to *Actinomyces.*

^*∗∗*^ND = not disclosed.

**Table 6:** Publications of cross-sectional studies of pelvic actinomycosis.

Sample size	Period	Age	Diagnosis	Diagnosis method	Previous use of IUD	Important findings	Reference
121,193	March 1977–November 1979	21–51	MSA^*∗*^	Papanicolaou	Yes = 11,952 (6 months–12 years)	202 cases with MSA^*∗*^, 2 patients were not IUD users	1980, Fry et al. South Africa [64]

2290	ND^*∗∗*^	17–76	MSA	Papanicolaou	Yes (prolonged use)	19 out of 2290 were diagnosed with MSA Statistically significant correlation of the presence of MSA with *Trichomonas vaginalis, cocci, lactobacilli*, pseudoeosinophils, endocervical cells, and polymorphs	2005, Demirezen et al. Turkey [65]

ND	January 1994–January 2010	6–75	Actinomycosis	TAC first and later histological report with finding of MSA	Yes = 2	23 cases of abdominal pelvic actinomycosis were identified: 18 women: 5 had ovarian and pelvic masses, 2 in the uterus; as an important risk factor, 2 patients used IUDs	2011, Sung et al. Korea [66]

293	March 1978–March 1979	ND	MSA	Papanicolaou sample observations	Yes = 128 plastic IUDs and 167 copper IUDs Oral contraceptives = 300	40 women with IUDs had increased prevalence of MSA, 2 who used copper and none who used oral contraceptives	1980, Duguid et al. England [67]

468 Comparative study with 4 sample groups	ND	33 ± 7.7	MSA	Papanicolaou samples	Cases without IUD (group 0: 128) 2 to 35 months IUD use (group 1: 121); 36 to 71 months (group 2: 112); more than 72 months (group 3: 107)	MSA was found in 2 cases from group 2 and in 7 patients from group 3 MSA was 3.68 times more likely with greater use	1999, Garrido et al. Colombia [68]

1774	January 1996–January 2001	22–51	Actinomycosis	Papanicolaou samples	Yes = 671 oral contraceptive methods = 343 Other contraceptive methods = 32 No contraceptive use = 728	Actinomycosis in 13 patients with IUDs and in 2 without contraceptive methods	2002, Torres et al. Chile [69]

22		24–58	Genital actinomycosis	Biopsy results	Yes = 18 (3–19 years)		2003, Madrid et al. Chile [70]

200	ND	25–50	*Actinomyces*	Vaginal secretion culture, Gram stain, Papanicolaou, API 20A biochemical assays	Yes = 106 (3–10 years) No = 94	*Actinomyces* in 14 patients with IUDs, of which 2 had no symptoms of infection; species: *Actinomyces israelii, Actinomyces naeslund ii*, and* Actinomyces odontolyticus*	2002, Cano Ramos et al. Mexico [71]

^*∗*^MSA = microorganisms similar to *Actinomyces.*

^*∗∗*^ND = not disclosed.
